# Trimetazidine improves angiogenesis and tissue perfusion in ischemic rat skeletal muscle

**DOI:** 10.3389/fphar.2024.1436072

**Published:** 2024-07-23

**Authors:** Yongting Pan, Li Mai, Wenkai He, Xuqi Yang, Enting Wu, Jiayuan Zhao, Bailiang Liu, Mingyan Li

**Affiliations:** ^1^ Nanshan School, Guangzhou Medical University, Guangzhou, China; ^2^ Department of Cardiology, Guangzhou Institute of Cardiovascular Disease, Guangdong Key Laboratory of Vascular Diseases, The Second Affiliated Hospital, Guangzhou Medical University, Guangzhou, China; ^3^ The Second School of Clinical Medicine, Guangzhou Medical University, Guangzhou, China

**Keywords:** trimetazidine, angiogenesis, tissue perfusion, peripheral artery disease, HIF-1α

## Abstract

**Introduction:** Peripheral artery disease (PAD) is an increasingly common disease, causing significant complications for patients. Trimetazidine (TMZ) not only improves clinical symptoms in PAD patients but also facilitates angiogenesis in ischemic hind limbs. Our aim was to find the function of TMZ in promoting angiogenesis and tissue perfusion in ischemic rat skeletal muscle.

**Methods:** The rats underwent femoral artery ligation (FAL) and then treated with TMZ and saline. Hematoxylin-eosin and Masson’s trichrome stain in the ischemic gastrocnemius muscle to analyze muscle morphology and atrophy. To identify angiogenesis and the tissue perfusion, CD31 immunohistochemical staining and laser speckle contrast imaging was conducted. Additionally, hind limb motor ability was measured. Finally, qRT-PCR and Western blotting were used to statistically analyze the expression levels of HIF-1α and VEGF.

**Results:** Our study demonstrated significant enhancement in angiogenesis and tissue perfusion after FAL when treated with TMZ compared to the saline group. Histologically, it mitigates ischemia-induced muscle atrophy and inflammation, as well as reduces fibrosis progression in the TMZ group. Additionally, hind limb motor ability improved in rats treated with TMZ during motor experiments.

**Discussion:** It suggests that TMZ can promote angiogenesis and improve tissue perfusion in ischemic skeletal muscle of rats by activating the HIF-1α/VEGF signaling pathway. Additionally, it leads to significant improvement in ischemia-induced motor limitations in the hind limbs of rats.

## 1 Introduction

Peripheral artery disease (PAD) has emerged as a significant health concern among the elderly population in recent years, presenting a diverse range of clinical manifestations (Song et al., 2019; Polonsky and McDermott, 2021). Complications associated with PAD, such as severe limb ischemia and lower limb amputation, not only pose devastating consequences for patients, but also contribute to the growing burden on healthcare systems (Criqui et al., 2021). Atherosclerotic plaques leading to arterial stenosis or occlusion are recognized as the primary cause of blood flow obstruction, particularly in the lower extremities (Crawford et al., 2016). Ischemic events in the legs and feet subsequently further exacerbate myopathy development and tissue necrosis. And then the gangrene or ischemic ulcers may ensue when there is severe ischemia, (Golledge, 2022). Despite significant advancements in surgical and pharmacological interventions for PAD, such as gene-based, cell-based and small-molecule therapies, many patients, especially those in the advanced stages of the disease, have shown only limited effectiveness from these therapies.

Ischemia-induced angiogenesis occurs rapidly when the body experiences ischemia. And the hypoxic skeletal muscle cells respond to ischemia by secreting multiple factors, including HIF-1α, ANG-1, VEGF, and PDGF-BB (Richardson et al., 1995). HIF-1α, as a pivotal oxygen-sensitive subunit, plays a crucial role in regulating hypoxic-ischemic responses (Berra et al., 2003). Experimental evidence supports that HIF-1α promotes VEGF expression and CD31-positive capillary formation in skeletal muscle (Niemi et al., 2014). VEGF also plays a critical role in regulating angiogenesis (Apte et al., 2019). Under hypoxic conditions, upregulated VEGF dominantly drives angiogenesis by activating receptor signaling on endothelial cells (Ameln et al., 2005).

Trimetazidine (TMZ) is an approved antianginal medication with metabolic properties (Montalescot et al., 2013). It improves ischaemic myocardial energy in cardiovascular disease by directly inhibiting long-chain 3-ketoacyl-coenzyme A thiolytic enzyme activity, and then sustaining ATP production with less oxygen demand (Kantor et al., 2000). Multiple randomized controlled trials have confirmed trimetazidine’s protective effect against heart failure (Tuunanen et al., 2008; Fragasso et al., 2011), establishing its unique position in cardiovascular therapy. Furthermore, TMZ exerts its effects not only on cardiomyocytes (Zhao et al., 2019), but exhibits antifibrotic and protective properties on non-cardiomyocytes (Singh and Chopra, 2004). In the clinical setting, comparing exercise training alone *versus* combined with TMZ adjuvant therapy in patients with PAD and claudication, it was found that adding TMZ significantly improved maximum walking distance (Vitale et al., 2011). Additionally, another research incorporated TMZ into conventional therapy and found that it enhanced both the distance and duration of walking in patients to their maximum capacity (Chu et al., 2016).

In laboratory experiments, pre-treatment of rat mesenchymal stem cells with TMZ not only significantly protects against H2O2-induced loss of cell viability and oxygen metabolism, but also results in a substantial increase in the level of HIF-1α (Wisel et al., 2009). Furthermore, intervention with TMZ can upregulate the content of HIF-1α in rats undergoing renal ischemia-reperfusion (Mahfoudh-Boussaid et al., 2014). Consider the findings above, TMZ may be a candidate for replacement of conventional agents in the treatment of angiogenesis. However, the impact of TMZ on angiogenesis and tissue perfusion in ischemic hind limb models has not yet been revealed. Moreover, no study to date has examined the mechanism by which TMZ regulates angiogenesis in the gastrocnemius muscle. In this study, hindlimb ischemia was induced in Wistar rats and interventions with TMZ and saline were administered to investigate its effects. The ischemic gastrocnemius muscle was stained with hematoxylin-eosin (H&E) and Masson’s trichrome to qualitatively analyze muscle morphology and atrophy. Subsequently, angiogenesis was quantitatively evaluated by CD31 immunohistochemical staining. In addition, the tissue perfusion was evaluated by using laser speckle contrast imaging (LSCI) at different time points. Finally, qRT-PCR and Western blotting techniques were used to assess and statistically analyze the expression levels of HIF-1α and VEGF. The study aimed to figure out the impacts of TMZ on angiogenesis and tissue perfusion after hind limb ischemia in Wistar rats and to explore its possible mechanisms with Hif-1a/VEGF.

## 2 Materials and Methods

### 2.1 Animals

Adult male Wistar rats were obtained from Guangzhou Ruige Biological Technology Company Limited. All Wistar rats care and experimental procedures were granted approval by the Ethics Committee of Guangzhou Medical University (GY 2023-316, Guangzhou, China). All techniques employed in experiments and practices for animal care adhered to the regulations made by China’s Institutional Animal Care and Use Committee. Wistar rats, weighing between 200–250 g, were housed in a room with a circumambient temperature of around 25°C and a 12-h dark/light cycle. They were acclimatized to the laboratory for 1 week before the studies.

### 2.2 Experimental model

The Pentobarbital sodium salt (C11H17N2NaO3, purity >95%) was obtained from Nacalai Tesque, Inc. (Kyoto, Japan). Before anesthesia, we carefully check the effective use date of the anesthetic. The rats received intraperitoneal injections of 2% Pentobarbital sodium at a dosage of 50 mg/kg (dissolved in 0.9% NaCl) to induce anesthesia. During the experiment, we continuously monitored the animals in real-time. They were fully anesthetized before any procedures began. If there was any response to pain or stimulation, we postponed the operation until the animal no longer showed signs of pain under anesthesia. The body’s core temperature was maintained at a constant 37°C. A surgical incision was made in the hindlimb to expose and carefully dissect the femoral artery on the left leg. Equidistant ligation of the femoral artery was performed using 7-0 silk sutures placed proximally and distally in all rats. Subsequently, excision of the ligated vessels took place (Limbourg et al., 2009).

### 2.3 Experimental groups

After undergoing femoral artery surgery, rats were randomly allocated into three groups. The control group (n = 4) had their gastrocnemius muscle on the operated side sampled 0 days post-surgery. The TMZ group (n = 8) received a daily intragastric administration of 10 mg/kg TMZ (T166941, aladdin). The saline group (n = 8) was administered an equivalent volume of 0.9% NaCl solution at the same time. On days 7 and 14, ischemic gastrocnemius muscle samples (n = 4, each group) were collected from the surgical side for each respective group. We euthanized the animals on days 0, 7, and 14 after surgery. Firstly, we injected a large amount of sodium pentobarbital (150 mg/kg) into the peritoneal cavity of the rat. We finally confirmed that there were no signs of breathing cessation and pain response by pressing their toes, before placing the rats in the freezer.

### 2.4 Laser speckle contrast analysis

After the rats were anesthetized, they were positioned in the prone position on a temperature-controlled plate for peripheral blood perfusion monitoring using LSCI (Pericam PSI, Perimed, Jarfalla, Sweden) (Gallego-Perez et al., 2017). The monitoring was conducted on the preoperative day, postoperative day 0, postoperative day 7, and postoperative day 14. The date of day 1 post-surgery is to confirm the presence of ischemia. According to the manufacturer’s instructions, the distance between the laser head and skin surface was fixed at 15.3 cm. The images were stored and analyzed offline on a computer with an image collection rate set to 10 times per second. A standardized circular region of interest (ROI) measuring 50 mm^2^ was selected in the middle of the plantar sole of the surgical side of the foot to represent average blood flow in perfusion units (PU). ROI can reduce spatial variability. Subsequently, perfusion analysis was then set to 60 s (also referred to as time of interest (TOI) by manufacturers). Due to the influence of periodic tissue movement, such as respiration in rats, we set TOI to reduce measurement variability when assessing tissue perfusion with LSCI (Zötterman et al., 2017).

### 2.5 The bar fatigue test

The bar fatigue test was employed to assess limb function (Han et al., 2021). In order to familiarize animals with the apparatus (Ugo Basile, Italy, Model 7750), they were acclimated 1 day before the test. On the testing day, rats were positioned on the apparatus and measurements commenced with a rotation speed of 0 rpm. Subsequently, the rotation speed was increased at a rate of 7.2 rpm per minute, and the time until rat fall-off was recorded. Each rat underwent three trials, separated by a 10-min interval between sessions. If any of these opportunities resulted in a rat successfully holding on for 5 min or more, it would be removed from the apparatus and its duration recorded as 300 s. The average of these three experimental durations was then calculated.

### 2.6 Histological analysis

The left hind gastrocnemius muscles were collected from euthanized rats, followed by rinsing with phosphate-buffered saline (PBS), fixation in 4% paraformaldehyde, and embedding in paraffin. Subsequently, the samples were sectioned at 4 µm using a microtome (RM 2016, Leica, Bensheim, Germany) for the histological analysis. HE staining (G1005, Servicebio, China) was used to observe the morphological changes of muscle fibers at different time points. For the muscle fiber cross-sectional area analysis, images were calculated using the ImageJ software (NIH Image, Bethesda, MD, United States) in five random regions of each section. The extent of fibrosis formation can be assessed through Masson’s trichrome staining (G1006, Servicebio, China) by calculating the percentage of fibrotic area relative to cross-sectional area. Microscopic images of regenerated tissues were captured using a digital slide scanner while ImageJ was utilized for morphological analysis.

### 2.7 Immunohistochemistry (IHC)

IHC staining was performed on gastrocnemius muscle tissue to evaluate angiogenesis, using Servicebio Technology (Wuhan, China). The tissue slices were first subjected to thermal repair with EDTA antigen repair solution after dewaxing. Subsequently, the slices underwent three washes with PBS solution and were then treated with 3% hydrogen peroxide to suppress natural peroxidase activity. Following this, the slices were coated with serum for 30 min and then exposed to anti-CD31 (GB113151, 1:600) overnight at 4°C. After rinsing, the secondary antibody (GB23303, 1:10000) was applied at 37°C for 30 min. Finally, the tissues underwent DAB chromogenic staining and hematoxylin restaining before dehydration, sealing, microscopic observation, and photography.

CD31 was utilized as a marker for endothelial cells, with its expression serving as an indicator of microvessel density. A single vascular endothelial cell positive for CD31 or an aggregate of such cells forming a cluster was designated as a vessel. Initially, the entire section was examined at low magnification (×100) to identify three areas exhibiting the highest microvessel density, commonly referred to as hot spots. Subsequently, microvessels labeled with the CD31 antibody were quantified under high magnification (×200) (Chen et al., 2020). Microvessel density was expressed as the number of CD31-positive cells per square millimeter. Quantitative analysis of microvessel density (MVD) was conducted using ImageJ software.

### 2.8 Western blotting analysis

After quantifying the proteins using the BCA method, 13 μL of protein samples were extracted from rat gastrocnemius lysate. These samples were then separated by 10% sodium dodecyl sulfate polyacrylamide gel electrophoresis (SDS-PAGE) and transferred onto polyvinylidene fluoride membranes (PVDF). Initially, the membranes underwent a 2-h blocking process using a solution of Tris buffer saline with Tween-20, which included 5% fat-free dry milk. Subsequently, the membranes were incubated with primary and secondary antibodies. We used HIF-1α (Cat#14179, Cell Signaling Technology), GAPDH (Cat#2118, Cell Signaling Technology), and VEGF (SC-7269, Santa Cruz) as our primary antibodies. As for secondary antibodies, goat anti-mouse IgG IRDye800 (Cat. No. 926-32210; Li-Cor) and goat anti-rabbit IgG IRDye680 (Cat. No. 926-68021; Li-Cor) were employed. GAPDH protein was used as a control for protein loading to confirm consistent protein loading in all analyzed samples. The bands were visualized using a chemical fluorescent reagent box (Bio-Rad, China) and an imaging system (Bio-Rad, China). The relative expression of these proteins was quantified after determining their gray values through ImageJ software analysis. A bar diagram is presented to illustrate quantified results obtained from at least three independent experiments.

### 2.9 Extraction of RNA and qRT-PCR

The TRIzol reagent (Life, USA) was employed to isolate total RNA from the left gastrocnemius muscle tissue. Following the instructions provided by the manufacturer, equal quantities of RNA were reverse transcribed into cDNA using the PrimeScript RT Master Mix (TakaRa, Japan). Gene expression levels of HIF-1α, VEGF, and GAPDH were quantified by qPCR in a 20 μL reaction on the ABI-7500 StepOneTM Real-Time PCR instrument (Thermo, USA) using the TB GreenTM Premix Ex TaqTM II Reactors Box (Takara, Japan) in accordance with the manufacturer’s protocol. GAPDH was used as a reference gene and relative mRNA expression was calculated using the 2^−ΔΔCT^ method. Primer sequences were as follows:

GAPDH-F: 5′- GTG​CCA​GCC​TCG​TCT​CAT​A,

GAPDH-R: 5′- GTT​GAA​CTT​GCC​GTG​GGT​AG;

HIF-1α-F: 5′-AGC​AAT​TCT​CCA​AGC​CCT​CC;

HIF-1α-R: 5′-TTC​ATC​AGT​GGT​GGC​AGT​TG;

VEGF-F: 5′-GGG​AGC​AGA​AAG​CCC​ATG​AA,

VEGF-R: 5′- AGA​TGT​CCA​CCA​GGG​TCT​CA.

### 2.10 Statistical analysis

The mean ± standard error of the mean (SEM) or median (95% CI) were used to present the data. Statistical analysis was conducted using GraphPad Prism 5.0 software (GraphPad Software, San Diego, CA, United States). The Student’s t-test was used to compare two groups, while the one-way ANOVA and Tukey’s honestly significant difference test were used for comparing multiple groups. A *p*-value below 0.05 was considered statistically significant. All experiments were replicated at least three times.

## 3 Result

### 3.1 TMZ slows down muscle atrophy and fibrosis in the ischaemic gastrocnemius of Wistar rats

**FIGURE 1:**
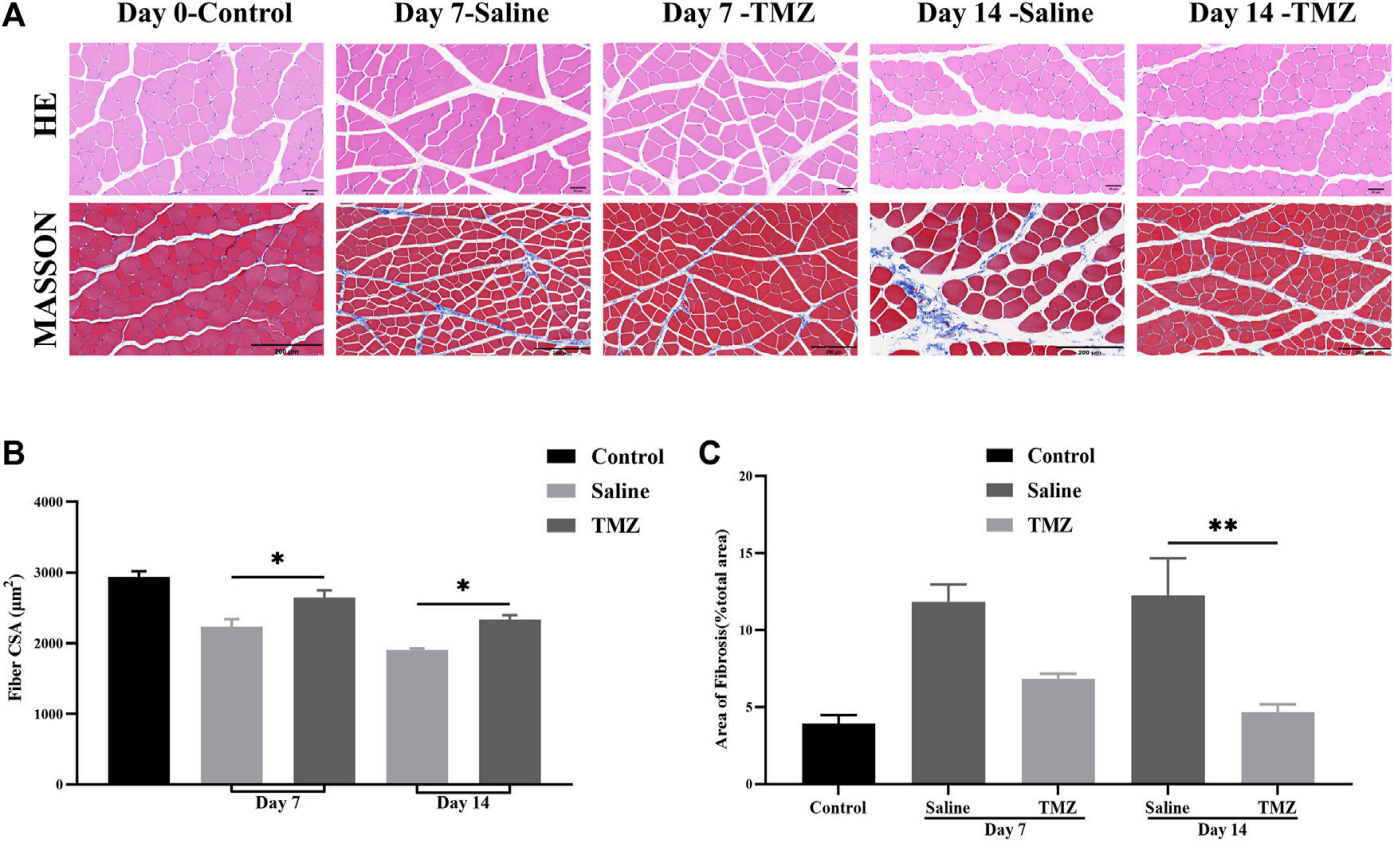
TMZ slows down muscle atrophy and fibrosis in the ischaemic gastrocnemius of Wistar rats (n = 4, each group at each time point). **(A)** Representative haematoxylin and eosin (HE) staining (400X, Scale bar = 50 μm) and Masson’s staining (200X, Scale bar = 200 μm) in ischaemic gastrocnemius muscles sections of Wistar rats. **(B)** Muscle fiber cross-sectional area (CSA) in gastrocnemius muscles of rats. **(C)** Quantitative analysis of the fibrotic area in Masson’s staining sections. The values are expressed as mean ± standard error of the mean (SEM). *p < 0.05, **p < 0.01. TMZ, trimetazidine. MASSON, masson’s trichrome staining.

Skeletal muscle ischemia induced by femoral artery ligation is manifested by monocyte infiltration and muscle atrophy (Figure 1A). The skeletal muscles of the control group showed normal histological structure. With the increase of time, monocyte infiltration was obvious in both the saline group and the TMZ group, but the situation in the TMZ group was alleviated. Compared with the TMZ group (Figure 1B), the muscle fiber cross-sectional area (CSA) was slightly reduced in the saline group, both on the 7th postoperative day (*p* < 0.05) and the 14th postoperative day (*p* < 0.05). These results suggest that TMZ therapy alleviates ischemia-induced muscle atrophy and inflammation.

Following masson’s trichrome staining, the analysis revealed a significant impact of TMZ and saline on fibrosis levels in the ischemic gastrocnemius muscle (Figure 1A). Both groups showed increased collagen fiber content within the muscle tissue on day 7 after femoral artery ligation (FAL). However, at 14 days post-surgery, the fibrotic area was notably lower in the TMZ group compared to the saline intervention (4.60% ± 0.50% vs. 12.30% ± 2.40%; *p* < 0.01). Additionally, there was a progressive decrease in fibrosis over time observed in the TMZ group (Figure 1C).

### 3.2 TMZ improves tissue perfusion in the ischaemic lower limb of Wistar rats

**FIGURE 2:**
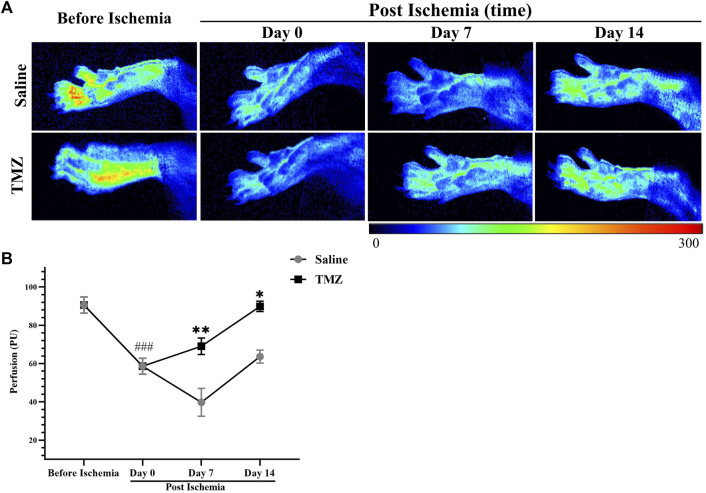
TMZ significantly promotes perfusion recovery in rats with ischemic hind limbs. **(A)** Representative tissue perfusion images of each group at various time points. Results of the LASCA in two groups of experimental rats (n = 4, each group). Compared with before ischemia (n = 8), the animals showed significantly reduced tissue blood flow on day 0 post ischemia (n = 8), indicating that the model was successfully constructed. The results showed that TMZ promoted blood perfusion from day 0 to day 14 post ischemia. **(B)** Values are expressed as average ± SEM. *p < 0.05, **p < 0.01 *versus* corresponding the saline group; ###*p* < 0.001, day 0 post ischemia *versus* before ischemia; TMZ, trimetazidine.

To assess changes in peripheral perfusion, we compared the ischemic limbs at different time points: preoperatively, immediately postoperatively, and 7 and 14 days postoperatively. Figure 2A displays all tissue perfusion images, revealing significant enhancements in flow over time with TMZ as compared to saline. After conducting statistical analysis on the average PU in the ROI, we observed significant differences in blood flow analysis between the two groups before ischemia and day 0 post ischemia (*p* < 0.01), indicating successful establishment of the ischemic model. Additionally, we found that the blood perfusion in the saline group was decreased continuously from 0 to 7 days post ischemia. In contrast, TMZ reversed the decrease in blood flow perfusion, suggesting that TMZ can promote blood flow recovery in ischemic lower limbs. Besides, the blood perfusion of TMZ increased continuously from 0 to 14 days post ischemia (Figure 2B), which indicated that TMZ was effective and prolonged.

### 3.3 TMZ significantly improves hindlimb motor ability of Wistar rats

**FIGURE 3:**
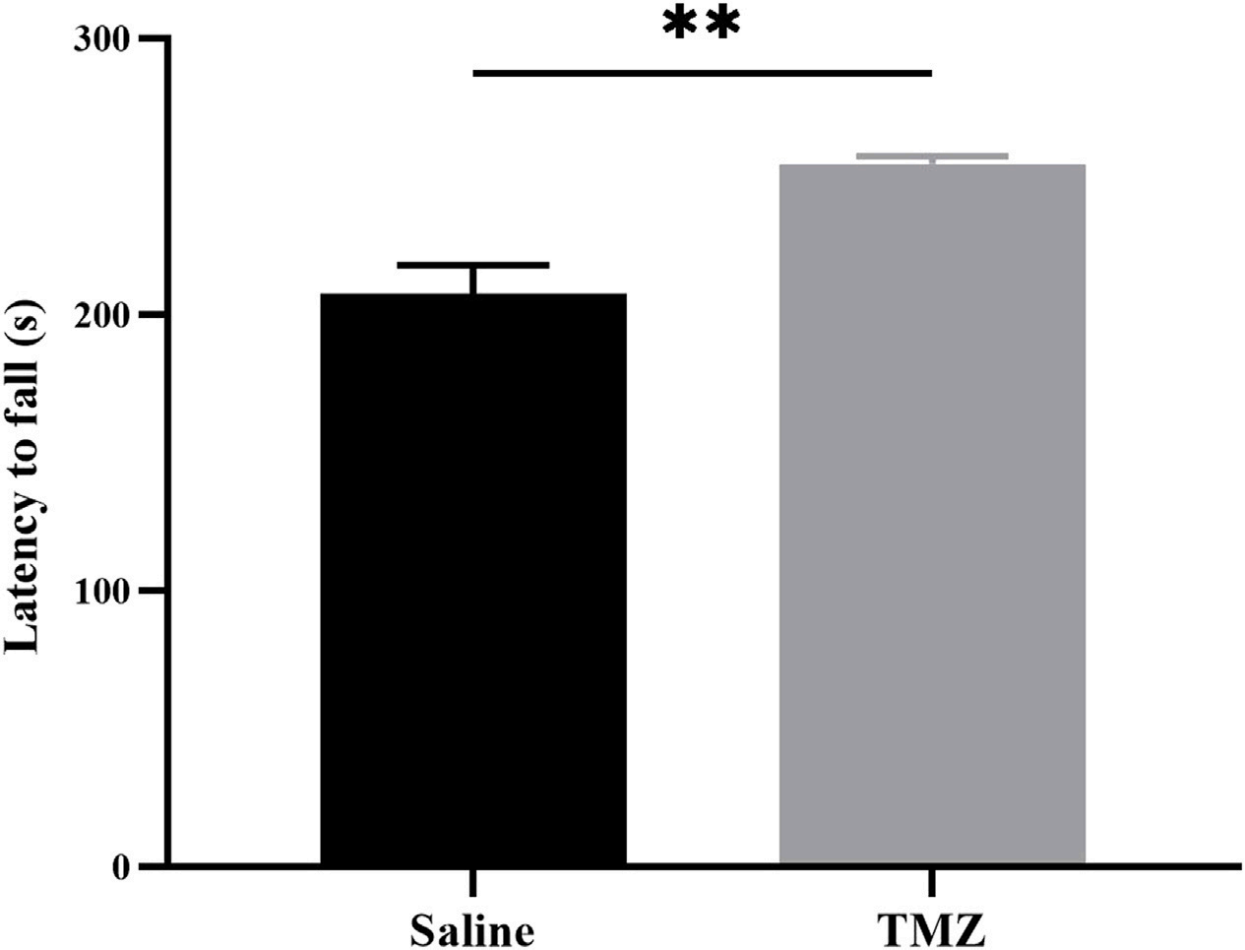
TMZ improves hindlimb motor performance in rats with ischemic hind limbs at the 14-day post-surgery time point. Results of the hindlimb motor performance in two groups of experimental rats (n = 4, each group). The values are expressed as mean ± SEM. *p < 0.05; TMZ, trimetazidine.

To evaluate hindlimb motor performance in Wistar rats across various experimental groups, we utilized the rotating bar fatigue apparatus. Following an acclimatization period, it was observed that the TMZ group exhibited a significantly prolonged holding time compared to the saline group (*p* < 0.05) (Figure 3). Based on these findings, it suggests that administration of TMZ effectively enhances motor performance in Wistar rats over a 2-week duration.

### 3.4 TMZ stimulates ischemia-induced angiogenesis in Wistar rats

**FIGURE 4:**
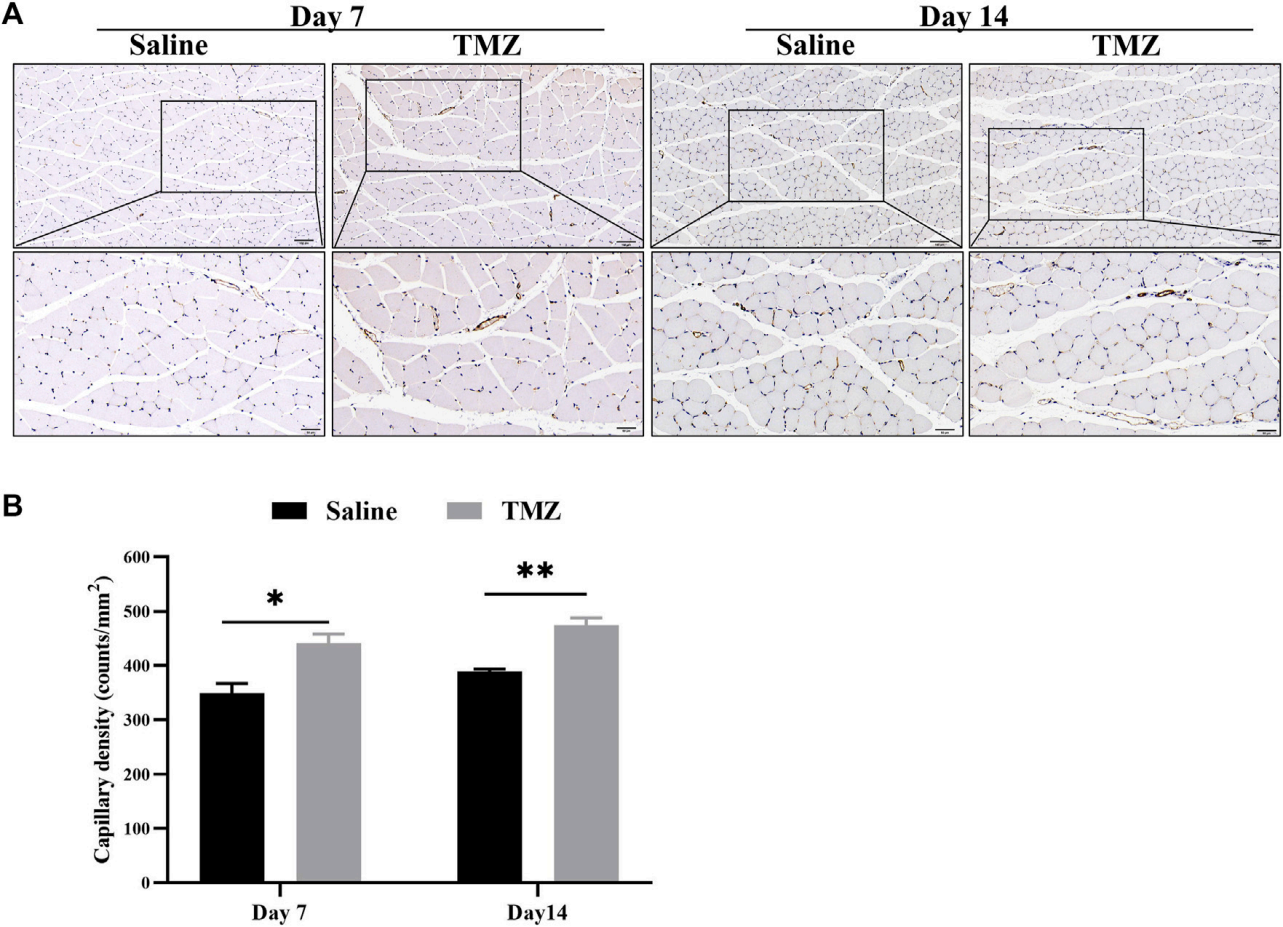
TMZ stimulates ischemia-induced angiogenesis in Wistar rats. **(A)** IHC staining of CD31 marker is shown (n = 4, each group at each time point). Microvessels were stained with CD31 antibody for immunohistochemistry. Brown spots show presence of endothelial cells (200X and 400X, Scale bar = 100 μm/50 μm). **(B)** Quantification of the microvessel density, which was expressed as the number of CD31-positive cells per square millimeter. Data is expressed as mean ± SEM. *p < 0.05, **p < 0.01; TMZ, trimetazidine.

The IHC staining results showed a progressive increase in the number of CD31-positive endocrine cells in both the TMZ-treated and saline groups, with a higher cell count observed in the TMZ-treated group compared to the saline group (Figure 4A) The group treated with TMZ demonstrated a gradual increase in capillary density over time, with statistically significant improvements observed at day 7 (*p* < 0.05) and day 14 (*p* < 0.01) (Figure 4B). These findings suggest that a 2-week intervention of TMZ significantly enhances angiogenesis in the ischemic limb compared to the saline group.

### 3.5 TMZ significantly activated HIF-1α/VEGF path in the ischaemic gastrocnemius of Wistar rats

**FIGURE 5:**
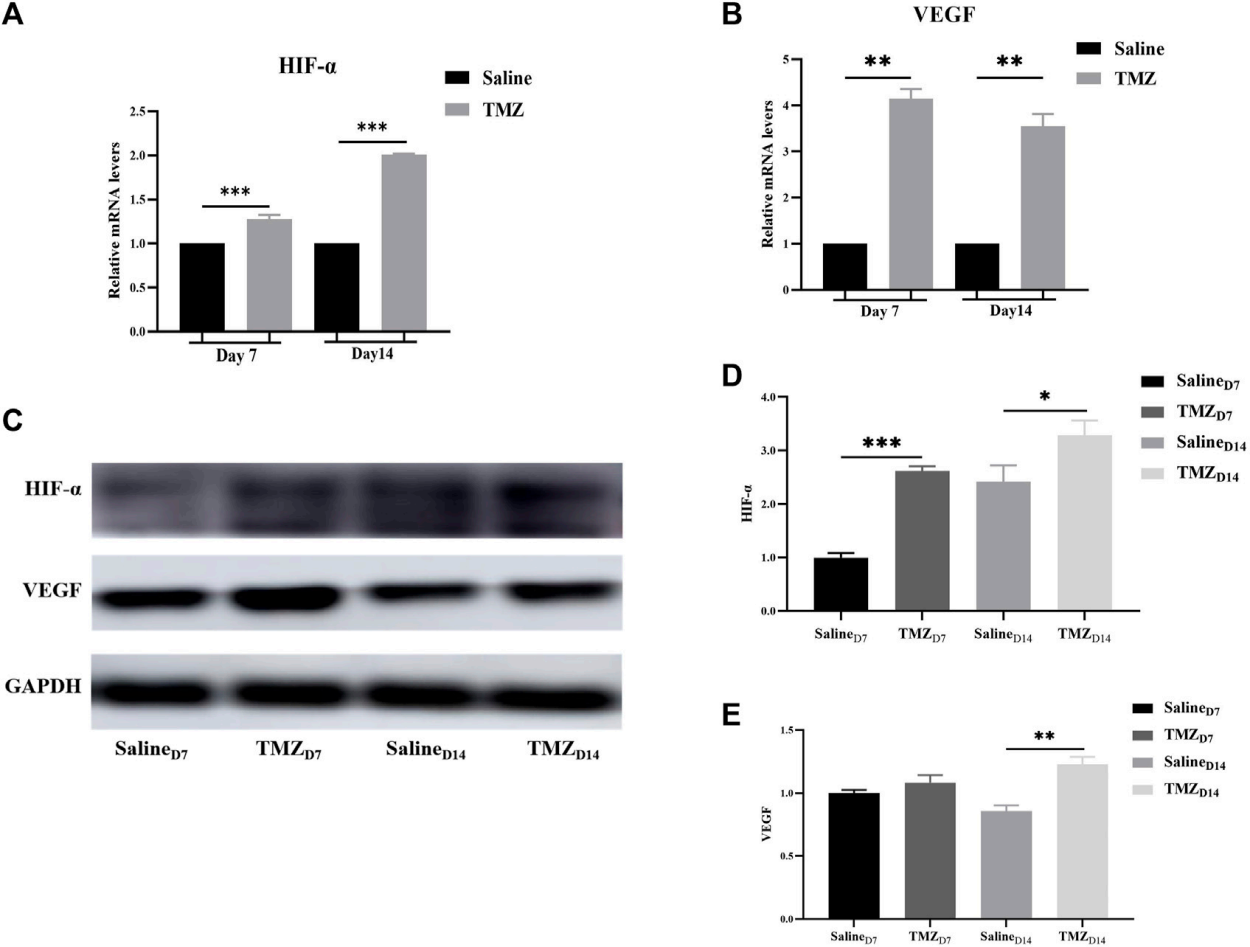
TMZ activates the HIF-1α/VEGF path in the ischaemic gastrocnemius of Wistar rats. **(A, B)** mRNA levels of HIF-1α and VEGF were detected by quantitative real-time PCR in different gastrocnemius tissues. **(C)** Western blot was used to analyze the protein expression of HIF-1α and VEGF in the saline and TMZ groups at 7 and 14 days after operation. **(D, E)** Quantitative analysis of HIF-1α and VEGF protein from Western blot results. Data are mean ± SEM. *p < 0.05; **p < 0.01; ***p < 0.001; HIF-1α, hypoxia inducible factor-1α; VEGF, vascular endothelial growth factor; TMZ, trimetazidine.

It is widely acknowledged that HIF-1α and VEGF are pivotal factors in the regulation of endothelial cell-mediated angiogenesis. To further elucidate the impact of TMZ on these key regulators, qRT-PCR and Western blot analyses were conducted at one and 2 weeks post-surgery. Compared to the saline group, there was a significant upregulation in mRNA levels of HIF-1α and VEGF on days 7 and 14 after surgery (Figures 5A, B). Consistent findings were observed for protein expression levels of HIF-1α and VEGF in ischemic gastrocnemius tissue (Figures 5C–E). In conclusion, these results provided evidence that TMZ significantly activated HIF-1α/VEGF path in the ischaemic gastrocnemius of Wistar rats.

## 4 Discussion

In the foreseeable future, there is an expected rise in the incidence of peripheral arterial disease, particularly in countries with lower and moderate incomes, due to environmental changes and unhealthy lifestyle habits (Song et al., 2019). Currently, there is a lack of therapeutic interventions capable of directly improving tissue perfusion in patients with PAD (Kullo and Leeper, 2015). Consequently, there is a demand for an innovative and safe treatment.

In addition to being a conventional antianginal drug, TMZ exhibits promising potential in the treatment of PAD (Vitale et al., 2011). Here, we demonstrate the therapeutic effect of TMZ in a rat model of hindlimb ischemia. Specifically, our findings indicate that: 1) TMZ had better performance in restoring tissue perfusion and angiogenesis in the ischemic hindlimb than the control group; 2) TMZ promoted angiogenesis and restored tissue perfusion by activating the HIF1α-VEGF signaling path and 3) TMZ reduced the degree of muscle injury and improved motor function in the ischemic limb. A previous study showed that overexpression of HIF-1 α increased the expression of multiple angiogenic genes in cultured endothelial cells, including VEGF, PLGF, PAI-1, and PDGF. In addition, they found that hif-1α overexpression improved tissue perfusion in a rabbit model of acute hindlimb ischemia (Li et al., 2011). However, to the best of our knowledge, no studies have explored the mechanism by which TMZ promotes angiogenesis and tissue perfusion through HIF-α. Therefore, we elucidate the mechanism of TMZ on HIF-α in a rat model of hind limb ischemia, which is the first to be elucidated.

In our study, we performed FAL on Wistar rats and successfully established an animal model of hind limb ischemia by measuring peripheral perfusion before and after surgery, accurately mimicking the impaired vascular conditions in patients with PAD. Treatment strategies for ischemic diseases are mainly aimed at optimizing blood flow and alleviating ischemic areas (Norgren et al., 2007). Despite there are surgical and pharmacological interventions for PAD, they have shown only limited effectiveness. Therefore we aimed to investigate the impact of TMZ on angiogenesis and tissue perfusion in lower limb ischemia. Firstly, compared to the saline group, TMZ intervention significantly increased CD31 density in the ischemic gastrocnemius muscle. It showed that TMZ can promote angiogenesis in skeletal muscle affected by ischemia. These results align with prior research indicating that TMZ treatment ameliorates foot injury and promotes angiogenesis in diabetic mice with ischemic hind limbs by modulating the expression of ICAM-1 and VEGF-A (Yang et al., 2021). Notably, our datas showed that peripheral blood flow rapidly recovered in the TMZ group on the 7th day after surgery, highlighting the potential therapeutic effect of TMZ in restoring tissue perfusion. Here, our study verified the efficacy of TMZ in restoring tissue perfusion and enhancing angiogenesis in ischemic limb.

It is widely recognized that the hypoxic environment after ischemia is the main stimulus factor for angiogenesis, and HIF-1α, as a key regulator of hypoxic-ischemic response, plays a crucial role in the ischemic hypoxia response (Semenza, 2012). In hypoxic environments, HIF-1α escapes proteasome breakdown and combines with HIF-1β to form a complex, which recognizes the hypoxia response element to facilitate transcription of target genes associated with HIF-1α. These target genes play crucial roles in various biological processes including apoptosis, cell proliferation, and angiogenesis (Plastino et al., 2021).

Previous reports showed that compared with no or low dose TMZ intervention, the high dose TMZ treatment group showed an increase in HIF-1α protein and reached a peak on the 7th day after porcine kidney ischemia and reperfusion induction (Cau et al., 2008). In another study, TMZ alleviated folate-induced acute kidney injury in mice mainly by stimulating HIF-1α/heme oxygenase-1 (HO-1) (Abdelrahman et al., 2022). This means that TMZ may have a potential role in stabilizing HIF-1α and increasing its transcriptional activity. When comparing the transcription and expression of HIF-1α in ischemic skeletal muscle between the TMZ group and the saline group, we clearly observed that the mRNA and protein levels of HIF-1α were upregulated in the TMZ group. This result confirmed that TMZ also has the effect of activating HIF-1α expression in ischemic skeletal muscle.

Additionally, we observed that TMZ treatment resulted in a substantial increase in both mRNA and protein levels of VEGF, a downstream target gene regulated by HIF-1α, when compared to the control group. As a pivotal regulator in angiogenesis, HIF-1α governs the expression of numerous angiogenic genes including VEGF, placental growth factor (PLGF), angiopoietin 1 (ANGPT1), and angiopoietin 2 (ANGPT2) (Kelly et al., 2003). Among these genes, VEGF can activate its own expression through overexpression of both HIF-1α and HIF-2α, thereby promoting the formation of CD-31 expressing capillaries *in vivo* (Niemi et al., 2014). Therefore, it can be concluded that TMZ facilitates angiogenesis by activating the HIF-1α/VEGF signaling pathway.

PAD-related limb ischemia induces pathological changes including hypoxia, inflammation, muscle atrophy and even necrosis in leg muscle tissue (McDermott et al., 2020). One study showed that TMZ administration resulted in higher expression of the slow myosin heavy chain subtype and an increase in the number of small-sized muscle fibers (Ferraro et al., 2016). Moreover, recent evidence suggests that TMZ may alleviate dexamethasone-induced skeletal muscle atrophy by activating the PI3K/AKT pathway and partially inhibiting NLRP3 (Wang et al., 2021). These are consistent with our findings that TMZ treatment was histologically observed to reduce ischais-induced reductions in muscle fiber CSA and monocytes. Masson staining showed that TMZ treatment significantly reduced collagen fiber synthesis. Notably, after 2 weeks of treatment, we observed a significant improvement in movement restriction caused by posterior limb ischemia in TMZ treated rats. Therefore, in addition to increasing microvessel density to improve ischaemia induced movement restriction, TMZ may also relatively improve movement capacity by reducing inflammation, fibrosis, and muscular atrophy.

However, there are still certain limitations associated with this study. The TMZ, as a metabolic regulator, may potentially impact the energy metabolism of skeletal muscle. For instance, we have not yet investigated whether the enhancement of exercise capacity is also associated with the influence of TMZ on energy metabolism. However, further studies are required to validate this hypothesis. In conclusions, our study provides substantial evidence for the therapeutic efficacy of TMZ in promoting angiogenesis and enhancing peripheral perfusion through activation of the HIF-1α/VEGF signaling pathway within the ischemic limb. Moreover, it not only exerts a promotive influence on reducing ischemic induced muscle atrophy and inflammation, but exhibits anti-fibrotic properties in ischemic skeletal muscle. Additionally, hind limb motor ability improved in rats treated with TMZ during motor experiments. These findings offer novel insights into utilizing TMZ as a promising therapeutic strategy for PAD treatment.

## Data Availability

The original contributions presented in the study are included in the article/Supplementary Material, further inquiries can be directed to the corresponding author.

## References

[B1] Abdelrahman R. S. Abdelsalam R. A. Zaghloul M. S. (2022). Beneficial effect of trimetazidine on folic acid-induced acute kidney injury in mice: role of HIF-1α/HO-1. J. Biochem. Mol. Toxicol. 36 (5), e23011. 10.1002/jbt.23011 35191561

[B2] Ameln H. Gustafsson T. Sundberg C. J. Okamoto K. Jansson E. Poellinger L. (2005). Physiological activation of hypoxia inducible factor-1 in human skeletal muscle. Faseb J. 19 (8), 1009–1011. 10.1096/fj.04-2304fje 15811877

[B3] Apte R. S. Chen D. S. Ferrara N. (2019). VEGF in signaling and disease: beyond discovery and development. Cell. 176 (6), 1248–1264. 10.1016/j.cell.2019.01.021 30849371 PMC6410740

[B4] Berra E. Benizri E. Ginouvès A. Volmat V. Roux D. Pouysségur J. (2003). HIF prolyl-hydroxylase 2 is the key oxygen sensor setting low steady-state levels of HIF-1alpha in normoxia. Embo J. 22 (16), 4082–4090. 10.1093/emboj/cdg392 12912907 PMC175782

[B5] Cau J. Favreau F. Tillement J. P. Lerman L. O. Hauet T. Goujon J. M. (2008). Trimetazidine reduces early and long-term effects of experimental renal warm ischemia: a dose effect study. J. Vasc. Surg. 47 (4), 852–860. 10.1016/j.jvs.2007.10.036 18280092

[B6] Chen X. Wang H. Jiang Y. Li J. Li N. Kong J. (2020). Neovascularization in carotid atherosclerotic plaques can be effectively evaluated by superb microvascular imaging (SMI): initial experience. Vasc. Med. 25 (4), 328–333. 10.1177/1358863x20909992 32303154

[B7] Chu Y. S. Li D. X. Zhang M. Jiang T. M. (2016). Trimetazidine hydrochloride as a new treatment for patients with peripheral vascular disease-an exploratory trial. Eur. Rev. Med. Pharmacol. Sci. 20 (1), 188–193. 26813473

[B8] Crawford F. Welch K. Andras A. Chappell F. M. (2016). Ankle brachial index for the diagnosis of lower limb peripheral arterial disease. Cochrane Database Syst. Rev. 9 (9), Cd010680. 10.1002/14651858.CD010680.pub2 27623758 PMC6457627

[B9] Criqui M. H. Matsushita K. Aboyans V. Hess C. N. Hicks C. W. Kwan T. W. (2021). Lower extremity peripheral artery disease: contemporary epidemiology, management gaps, and future directions: a scientific statement from the American heart association. Circulation 144 (9), e171–e191. 10.1161/cir.0000000000001005 34315230 PMC9847212

[B10] Ferraro E. Pin F. Gorini S. Pontecorvo L. Ferri A. Mollace V. (2016). Improvement of skeletal muscle performance in ageing by the metabolic modulator trimetazidine. J. Cachexia Sarcopenia Mus. 7 (4), 449–457. 10.1002/jcsm.12097 PMC4864287 27239426

[B11] Fragasso G. Salerno A. Lattuada G. Cuko A. Calori G. Scollo A. (2011). Effect of partial inhibition of fatty acid oxidation by trimetazidine on whole body energy metabolism in patients with chronic heart failure. Heart 97 (18), 1495–1500. 10.1136/hrt.2011.226332 21700755

[B12] Gallego-Perez D. Pal D. Ghatak S. Malkoc V. Higuita-Castro N. Gnyawali S. (2017). Topical tissue nano-transfection mediates non-viral stroma reprogramming and rescue. Nat. Nanotechnol. 12 (10), 974–979. 10.1038/nnano.2017.134 28785092 PMC5814120

[B13] Golledge J. (2022). Update on the pathophysiology and medical treatment of peripheral artery disease. Nat. Rev. Cardiol. 19 (7), 456–474. 10.1038/s41569-021-00663-9 34997200

[B14] Han K. Jin X. Guo X. Cao G. Tian S. Song Y. (2021). Nrf2 knockout altered brain iron deposition and mitigated age-related motor dysfunction in aging mice. Free Radic. Biol. Med. 162, 592–602. 10.1016/j.freeradbiomed.2020.11.019 33248265

[B15] Kantor P. F. Lucien A. Kozak R. Lopaschuk G. D. (2000). The antianginal drug trimetazidine shifts cardiac energy metabolism from fatty acid oxidation to glucose oxidation by inhibiting mitochondrial long-chain 3-ketoacyl coenzyme A thiolase. Circ. Res. 86 (5), 580–588. 10.1161/01.res.86.5.580 10720420

[B16] Kelly B. D. Hackett S. F. Hirota K. Oshima Y. Cai Z. Berg-Dixon S. (2003). Cell type-specific regulation of angiogenic growth factor gene expression and induction of angiogenesis in nonischemic tissue by a constitutively active form of hypoxia-inducible factor 1. Circ. Res. 93 (11), 1074–1081. 10.1161/01.Res.0000102937.50486.1b 14576200

[B17] Kullo I. J. Leeper N. J. (2015). The genetic basis of peripheral arterial disease: current knowledge, challenges, and future directions. Circ. Res. 116 (9), 1551–1560. 10.1161/circresaha.116.303518 25908728 PMC4410432

[B18] Li M. Liu C. Bin J. Wang Y. Chen J. Xiu J. (2011). Mutant hypoxia inducible factor-1α improves angiogenesis and tissue perfusion in ischemic rabbit skeletal muscle. Microvasc. Res. 81 (1), 26–33. 10.1016/j.mvr.2010.09.008 20937289

[B19] Limbourg A. Korff T. Napp L. C. Schaper W. Drexler H. Limbourg F. P. (2009). Evaluation of postnatal arteriogenesis and angiogenesis in a mouse model of hind-limb ischemia. Nat. Protoc. 4 (12), 1737–1746. 10.1038/nprot.2009.185 19893509

[B20] Mahfoudh-Boussaid A. Hadj Ayed Tka K. Zaouali M. A. Roselló-Catafau J. Ben Abdennebi H. (2014). Effects of trimetazidine on the Akt/eNOS signaling pathway and oxidative stress in an *in vivo* rat model of renal ischemia-reperfusion. Ren. Fail. 36 (9), 1436–1442. 10.3109/0886022x.2014.949765 25246344

[B21] McDermott M. M. Ferrucci L. Gonzalez-Freire M. Kosmac K. Leeuwenburgh C. Peterson C. A. (2020). Skeletal muscle pathology in peripheral artery disease: a brief review. Arterioscler. Thromb. Vasc. Biol. 40 (11), 2577–2585. 10.1161/atvbaha.120.313831 32938218 PMC9571495

[B22] Montalescot G. Sechtem U. Achenbach S. Andreotti F. Arden C. Budaj A. (2013). 2013 ESC guidelines on the management of stable coronary artery disease: the task force on the management of stable coronary artery disease of the European society of cardiology. Eur. Heart J. 34 (38), 2949–3003. 10.1093/eurheartj/eht296 23996286

[B23] Niemi H. Honkonen K. Korpisalo P. Huusko J. Kansanen E. Merentie M. (2014). HIF-1α and HIF-2α induce angiogenesis and improve muscle energy recovery. Eur. J. Clin. Invest. 44 (10), 989–999. 10.1111/eci.12333 25208310

[B24] Norgren L. Hiatt W. R. Dormandy J. A. Nehler M. R. Harris K. A. Fowkes F. G. (2007). Inter-society consensus for the management of peripheral arterial disease (TASC II). Eur. J. Vasc. Endovasc. Surg. 33 (Suppl. 1), S1–S75. 10.1016/j.ejvs.2006.09.024 17140820

[B25] Plastino F. Pesce N. A. André H. (2021). MicroRNAs and the HIF/VEGF axis in ocular neovascular diseases. Acta Ophthalmol. 99 (8), e1255–e1262. 10.1111/aos.14845 33729690

[B26] Polonsky T. S. McDermott M. M. (2021). Lower extremity peripheral artery disease without chronic limb-threatening ischemia: a review. Jama 325 (21), 2188–2198. 10.1001/jama.2021.2126 34061140

[B27] Richardson R. S. Noyszewski E. A. Kendrick K. F. Leigh J. S. Wagner P. D. (1995). Myoglobin O2 desaturation during exercise. Evidence of limited O2 transport. J. Clin. Invest. 96 (4), 1916–1926. 10.1172/jci118237 7560083 PMC185828

[B28] Semenza G. L. (2012). Hypoxia-inducible factors in physiology and medicine. Cell. 148 (3), 399–408. 10.1016/j.cell.2012.01.021 22304911 PMC3437543

[B29] Singh D. Chopra K. (2004). Effect of trimetazidine on renal ischemia/reperfusion injury in rats. Pharmacol. Res. 50 (6), 623–629. 10.1016/j.phrs.2004.06.006 15501702

[B30] Song P. Rudan D. Zhu Y. Fowkes F. J. I. Rahimi K. Fowkes F. G. R. (2019). Global, regional, and national prevalence and risk factors for peripheral artery disease in 2015: an updated systematic review and analysis. Lancet Glob. Health 7 (8), e1020–e1030. 10.1016/s2214-109x(19)30255-4 31303293

[B31] Tuunanen H. Engblom E. Naum A. Någren K. Scheinin M. Hesse B. (2008). Trimetazidine, a metabolic modulator, has cardiac and extracardiac benefits in idiopathic dilated cardiomyopathy. Circulation 118 (12), 1250–1258. 10.1161/circulationaha.108.778019 18765391

[B32] Vitale C. Marazzi G. Pelliccia F. Volterrani M. Cerquetani E. Spoletini I. (2011). Trimetazidine improves exercise performance in patients with peripheral arterial disease. Pharmacol. Res. 63 (4), 278–283. 10.1016/j.phrs.2011.01.003 21220024

[B33] Wang L. Jiao X. F. Wu C. Li X. Q. Sun H. X. Shen X. Y. (2021). Trimetazidine attenuates dexamethasone-induced muscle atrophy via inhibiting NLRP3/GSDMD pathway-mediated pyroptosis. Cell. Death Discov. 7 (1), 251. 10.1038/s41420-021-00648-0 34537816 PMC8449784

[B34] Wisel S. Khan M. Kuppusamy M. L. Mohan I. K. Chacko S. M. Rivera B. K. (2009). Pharmacological preconditioning of mesenchymal stem cells with trimetazidine (1-[2,3,4-trimethoxybenzyl]piperazine) protects hypoxic cells against oxidative stress and enhances recovery of myocardial function in infarcted heart through Bcl-2 expression. J. Pharmacol. Exp. Ther. 329 (2), 543–550. 10.1124/jpet.109.150839 19218529 PMC2672865

[B35] Yang Y. Xu Q. Li T. Shao S. (2021). Trimetazidine ameliorates hindlimb ischaemic damage in type 2 diabetic mice. Ann. Med. 53 (1), 1099–1107. 10.1080/07853890.2021.1925147 34259103 PMC8281072

[B36] Zhao Y. Li S. Quan E. Zhang H. Wu Y. Luo Y. (2019). Trimetazidine inhibits cardiac fibrosis by reducing reactive oxygen species and downregulating connective tissue growth factor in streptozotocin-induced diabetic rats. Exp. Ther. Med. 18 (2), 1477–1485. 10.3892/etm.2019.7705 31363380 PMC6614713

[B37] Zötterman J. Mirdell R. Horsten S. Farnebo S. Tesselaar E. (2017). Methodological concerns with laser speckle contrast imaging in clinical evaluation of microcirculation. PLoS One 12 (3), e0174703. 10.1371/journal.pone.0174703 28358906 PMC5373607

